# A rare case of benign isolated follicular mucinosis with significant eosinophilic infiltration

**DOI:** 10.1016/j.jdcr.2026.03.019

**Published:** 2026-03-19

**Authors:** Shuxian Guo, Handan Cao, LinLin Ma, Guan Jiang

**Affiliations:** a Department of Dermatology, Affiliated Hospital of Xuzhou Medical University, Xuzhou, China; b Department of Dermatology, Xuzhou Medical University, Xuzhou, China; c Department of Dermatology, The General Hospital of Xuzhou Mining Group, Xuzhou, China

**Keywords:** corticosteroid injection therapy, eosinophils, follicular mucinosis, histopathology, topical

## Case description

A 20-year-old male presented with a 3-month history of a raised plaque on the forehead. Approximately 3 months prior, an erythematous plaque had developed on the glabella without obvious cause. The lesion gradually became elevated with indistinct borders, accompanied by exudation, small vesicles, and crusting. It was firm and mildly tender to palpation. The patient had been evaluated at an outside hospital where a skin infection was diagnosed and treated with oral and topical antibiotics, with poor response.

**Fig 1:**
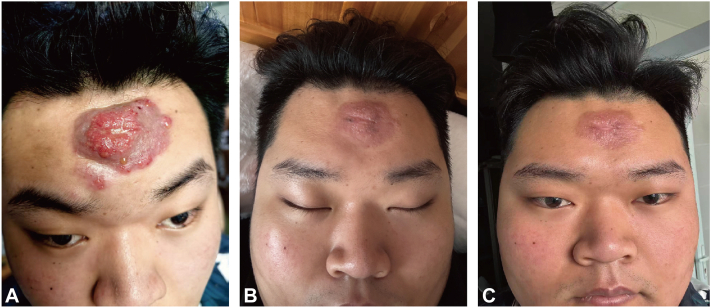
**A,** An egg-sized plaque with exudation on the forehead. **B,** Marked subsidence of inflammation after 1 month of treatment with oral methylprednisolone, isotretinoin, and hydroxychloroquine combined with topical pimecrolimus cream. **C,** Marked regression of the lesion after local injection of betamethasone.

**Fig 2:**
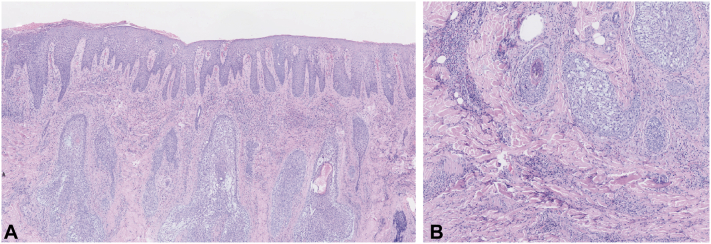
**A,** Spongiosis of the epidermis with parakeratosis and serous exudation. **B,** Dense eosinophilic infiltration with focal lymphocytic infiltration around small dermal vessels, around sweat glands, and between collagen bundles. Prominent mucin deposition was observed in the follicular epithelium in the dermis, without granuloma formation.

Examination at our institution revealed a 6.0 × 4.0 cm red, elevated plaque with exudation on the forehead (Fig 1, *A*). Histopathology demonstrated abundant mucin deposition within hair follicles and marked eosinophilic infiltration in the dermis (Fig 2). T-cell receptor (*TCR*) gene rearrangement studies and other laboratory workups were unremarkable.

The treatment regimen comprised oral corticosteroids, oral hydroxychloroquine, and isotretinoin, in conjunction with topical 0.1% pimecrolimus and fusidic acid ointment. After 10 days of therapy, the lesions showed significant reduction and exudation had resolved, although localized elevation persisted. By 1 month, inflammation had substantially subsided, leaving only residual elevation (Fig 1, *B*). Following intralesional injection of compound betamethasone, the lesions improved markedly. No recurrence was observed during follow-up (Fig 1, *C*).

**Question 1: What is the most likely diagnosis?**

**A.** Cutaneous infectious granuloma
**B.** Leprosy
**C.** Secondary infection of lichen planus
**D.** Folliculotropic mycosis fungoides
**E.** Primary benign idiopathic follicular mucinosis

**Answers:**

**A.** Cutaneous infectious granuloma – Incorrect. Cutaneous infectious granulomas generally occur in trauma-prone areas and are often preceded by a history of local injury. Histopathologically, they are characterized by well-formed inflammatory granulomas. Diagnosis is confirmed by the identification of a causative pathogen, typically through tissue culture or special stains for bacteria or fungi. The efficacy of targeted antimicrobial therapy provides further supportive evidence for the diagnosis.
**B.** Leprosy – Incorrect. Leprosy, caused by *Mycobacterium leprae*, can present with infiltrated plaques that may raise initial diagnostic consideration. However, it is distinguished by characteristic neurologic findings such as anesthesia or paresthesia within the lesions, which are absent in idiopathic follicular mucinosis. Histopathologically, leprosy demonstrates well-formed granulomas, often with perineural lymphoid infiltration, and acid-fast bacilli may be identified with Fite stain. Furthermore, a lack of response to antimycobacterial therapy would argue against leprosy in this context.^1^ In this case, histopathology did not reveal granulomatous changes, and antimycobacterial therapy was not administered.
**C.** Secondary infection of lichen planus – Incorrect. Although lichen planus can present with pruritic, violaceous papules or plaques that may become eroded or secondarily infected due to scratching, it is distinguished by its characteristic primary morphology and histopathology. Histopathologically, it features a dense band-like lymphocytic infiltrate at the dermo-epidermal junction, basal vacuolar degeneration, and necrotic keratinocytes (Civatte bodies).^2^
**D.** Folliculotropic mycosis fungoides – Incorrect. Secondary follicular mucinosis encompasses several categories, including lymphoma-associated follicular mucinosis, follicular mucinosis (FM) associated with other hematologic malignancies, and FM occurring in the context of non-hematologic diseases. Among these, folliculotropic mycosis fungoides (FMF) represents the most critical entity to exclude due to its overlapping histopathologic feature of follicular mucin deposition. However, FMF is characterized by a dense folliculotropic infiltrate of atypical lymphocytes with cerebriform nuclei, frequently forming Pautrier microabscesses. In this patient, *TCR* gene rearrangement studies were negative for a clonal T-cell population. This molecular finding, in conjunction with the absence of significant cytologic atypia on histopathology, effectively rules out FMF.^3^
**E.** Primary benign idiopathic follicular mucinosis – Correct. Primary benign idiopathic follicular mucinosis (PFM) is a rare histopathologic reaction pattern characterized by the accumulation of acid mucopolysaccharides within follicular epithelium and/or sebaceous glands, which stain positively with Alcian blue (pH 2.5). The condition predominantly presents in children and young adults, with a mean age of onset between 11 and 35 years. Histopathologic examination revealed prominent mucin deposition within the follicular epithelium, along with a dense eosinophilic infiltrate extending throughout the dermal layers. Scattered lymphocytes and eosinophils were also present around hair follicles. Notably, this eosinophil-rich pattern of infiltration supports a benign process. Additionally, *TCR* gene rearrangement studies showed no evidence of clonality, and no atypical cells were identified. Taken together, these findings are consistent with PFM.^4^

**Answer discussion**

FM is a rare dermatopathologic reaction characterized by the degeneration of follicular epithelium and the accumulation of mucin (primarily hyaluronic acid) within hair follicles, leading to their structural disruption. Clinically, it may present as papules, plaques, or alopecic patches. FM is broadly categorized into 2 types: PFM and secondary FM, the latter often associated with conditions such as mycosis fungoides.^5^ Benign, solitary cases with pronounced eosinophilic infiltration are exceptionally uncommon. In the present case, the patient’s young age, the frontal distribution of the lesions, the prominent eosinophilic infiltration observed histopathologically, and the overall clinical course collectively favored a diagnosis of primary benign idiopathic FM.

Most previously reported cases demonstrate mild or absent eosinophilic infiltration, with lesions generally presenting as follicular papules or plaques. The nonspecific clinical presentation in this patient could readily be misdiagnosed as early-stage cutaneous T-cell lymphoma (mycosis fungoides), fungal infection, other infectious granulomas, or eosinophilic pustulosis. For atypical or persistent lesions, dermatopathologic evaluation is recommended. When indicated, multiple biopsies from different sites may be performed, supplemented by T-cell immunohistochemical analysis to exclude FM associated with cutaneous T-cell lymphoma.^4^

Currently, no specific treatment exists for primary FM, and some cases may be self-limiting. The pathogenesis remains incompletely understood, with proposed mechanisms involving T-cell–mediated immune responses and cytokine-driven mucin deposition; eosinophilic infiltration may represent a reactive process secondary to excessive mucin production.^4^

Given the extensive cutaneous involvement, marked inflammation, and cosmetic concerns, the patient was treated with oral corticosteroids, hydroxychloroquine, and isotretinoin, along with intralesional compound betamethasone, resulting in marked clinical improvement and suggesting a possible adjunctive role for intralesional compound betamethasone in PFM.

In the present case, oral methylprednisolone in combination with hydroxychloroquine and isotretinoin, supplemented by intralesional compound betamethasone as needed, achieved marked clinical improvement. Nevertheless, long-term follow-up remains necessary to monitor for disease recurrence and potential malignant transformation.

## Conflicts of interest

None disclosed.
